# Complete Genome Sequence of *Spiroplasma citri* Strain R8-A2^T^, Causal Agent of Stubborn Disease in *Citrus* Species

**DOI:** 10.1128/genomeA.00206-17

**Published:** 2017-04-20

**Authors:** Robert E. Davis, Jonathan Shao, Yan Zhao, Gail E. Gasparich, Brady J. Gaynor, Nicole Donofrio

**Affiliations:** a Molecular Plant Pathology Laboratory, USDA, Agricultural Research Service, Beltsville, Maryland, USA; b College of Arts and Sciences, Salem University, Salem, Massachusetts, USA; c Department of Biological Sciences, Towson University, Towson, Maryland, USA; d Department of Plant and Soil Sciences, University of Delaware, Newark, Delaware, USA

## Abstract

*Spiroplasma citri* causes stubborn disease in *Citrus* spp. and diseases in other plants. Here, we report the nucleotide sequence of the 1,599,709-bp circular chromosome and two plasmids of *S. citri* strain R8-A2^T^. This information will facilitate analyses to understand spiroplasmal pathogenicity and evolutionary adaptations to lifestyles in plants and arthropod hosts.

## GENOME ANNOUNCEMENT

Citrus plants growing in arid and semiarid regions are affected by stubborn disease (citrus little leaf disease) (1–4). Symptoms include mottled and small leaves, abnormally shaped fruit, stunting of trees, and reduced production of fruits. Originally observed in California ca. 1915, the disease was first mistakenly attributed to infection by a virus (5, 6). Later, the disease was attributed to a (nonhelical) mycoplasma, based on results from electron microscopy (7, 8) that failed to reveal the three-dimensional structure of the disease agent. Two research groups subsequently reported *in vitro* culture of the presumed mycoplasma (9–13). Only after the discovery of a new form of cellular pathogen, that of helical and motile cell wall-less bacteria (14, 15), for which the name *Spiroplasma* was coined (16), was the citrus stubborn agent recognized to be a spiroplasma, and a new species was described, *Spiroplasma citri* (11, 17).

Thus far, a complete genome sequence has been reported for only one phytopathogenic spiroplasmal strain, that of *Spiroplasma kunkelii* CR2-3X (18). Here, we report the complete nucleotide sequence of the circular chromosome and two plasmids of *S. citri* strain R8-A2^T^ (Morocco-R8-A2 [ATCC 27556^T^]), which was originally isolated from stubborn diseased citrus [*Citrus sinensis* (L.) Osbeck var. Washington navel] (17). Genomic DNA was extracted from a culture of *S. citri* R8-A2^T^ grown in liquid medium, as described previously (19). Nucleotide sequencing was carried out using the next-generation sequencing (NGS) platform Pacific Biosciences (PacBio) single-molecule real-time (SMRT) sequencing system, in which 36,346 reads were obtained, totaling 420,842,286 nucleotides. The *N*_50_ read length was 16,325 nucleotides, the mean read length was 11,578 nucleotides, and the average reference consensus concordance was 100.00%. The assembled circular chromosome of 1,599,709 bp has an overall base composition of 25.56 mol% G+C; the average coverage per base position was 263.1×. The two plasmids were 26,182 and 14,987 bp in size and had base compositions of 22.79 and 24.63 mol% G+C, respectively. The assembled chromosome and plasmids were put through GeneMark.hmm (20) annotation and were curated by manual inspection using Artemis (21) as an annotation platform. The programs tRNAscan-SE 1.21 and RNAmmer (22) were used to predict regions encoding tRNAs and rRNAs, respectively. The chromosome has 1,573 protein-coding regions (CDSs), multiple insertions of spiroplasmavirus sequences, one set of rRNA genes, and 32 tRNA genes.

In addition to phytopathogens, non-plant-pathogenic spiroplasmas have been reported as transient residents on the surfaces of flowers, as symbionts of insects and ticks, as pathogens of crustaceans, and as a potential pathogen of humans (23–27). Phylogenetically, *S. citri* clusters with other cell wall-less bacteria in the class *Mollicutes* and is most closely related to plant pathogens *S. kunkelii* and *Spiroplasma phoeniceum*, and to the honey bee pathogen *Spiroplasma melliferum* (19, 28). The availability of the *S. citri* complete genome should facilitate studies to elucidate the evolutionary biology of plant-pathogenic spiroplasmas.

### Accession number(s).

This genome project has been deposited in GenBank under BioProject ID PRJNA296877, BioSample accession number SAMN04110376, and GenBank accession numbers CP013197 (chromosome), CP013198 (plasmid pSCI26), and CP013199 (plasmid pSCI15). The sequence versions described in this paper are CP013197.1, CP013198.1, and CP013199.1, respectively.
